# Paediatric Emergency Care in Low-Income Countries—Can Guidelines be Generalized?

**DOI:** 10.1177/2333794X241251787

**Published:** 2024-05-10

**Authors:** Abner Tagoola, Helena Hildenwall

**Affiliations:** 1 Jinja Regional Referral Hospital, Jinja, Uganda; 2 Department of Global Public Health, Karolinska Institutet, Stockholm, Sweden; 3 Astrid Lindgren Children’s Hospital, Karolinska University Hospital, Stockholm Sweden; 4 Department of Clinical Science, Intervention and Technology, Karolinska Institutet, Stockholm, Sweden

**Keywords:** children, emergency care, ETAT, guidelines, low-income countries

Paediatric in-hospital mortality rates remain high in low-income countries, with a significant proportion of deaths occurring within the first 24 hours of admission.^1^ Global and national health initiatives have primarily aimed at improving access to basic care in order to combat child mortality in these settings. Programs like The United Nations Children’s Fund (UNICEF) and World Health Organisation’s (WHO) Integrated Management of Neonatal and Childhood Illness (IMNCI)^2^ and the integrated Community Case Management (iCCM)^3^ have emphasized the early identification of suspected infections to facilitate prompt administration of treatment, such as antimalarials, antibiotics, oral rehydration solution, and zinc. Under these programs, children with severe symptoms should be referred to higher level care. However, challenges to attend referrals are common due to transportation difficulties and financial barriers. This may cause delays and lead to arrival at hospital in a late and more severe stage of illness, thereby putting high demands on an efficient emergency triage and assessment system, necessitating swift diagnosis and stabilization of critically ill children.

An estimated 60% of mortality in low-income countries is attributed to limitations in quality of care.^4^ Reports consistently highlight shortages in equipment and medicines, compounded by limited staff numbers amidst overwhelming patient loads. Moreover, the limited access to evidence-based guidelines and training for management of critically ill pediatric patients, exacerbates these gaps in care.

The well reported lack of diagnostic equipment and medicine in hospitals in low-income settings^5^ is an obvious and major limitation to quality care. Still, some improvements can be achieved even within existing systems. The Emergency Triage Assessment and Treatment (ETAT) was developed by the World Health Organization (WHO) in 2005 as an approach for hospitals in low income countries to rapidly assess and direct children to the most appropriate care.^6^ In the development of ETAT at a Malawian tertiary hospital, pediatric in-hospital mortality reduced from 10%-18% to 6%-8% after implementation.^7^ The ETAT training guidelines as currently provided by the WHO are brief and leave out some areas of importance for emergency care, such as cardio-pulmonary resuscitation, anaphylactic and diabetic shock, continued supportive care, and monitoring. ETAT updates were provided as an amendment in 2016,^8^ and include revisions related to recently released research reports, including consideration of fluid management.^9^ Still, the training material available online has not yet been updated or expanded and additional revisions may be needed to include updates for more acute conditions. Individual organizations in low-income countries, with the Kenyan and Rwandan Pediatric Associations as leading examples, have updated the originally developed ETAT guidelines to provide more detailed instructions on equipment and processes needed for implementation. These manuals offer a more comprehensive training package on the acute conditions that are commonly encountered among children in resource-constrained hospitals. This extended version of ETAT is known as ETAT Plus (ETAT+) and includes sections on inpatient care, including monitoring of fluid intake and continued care on the ward.

While improved adherence to clinical guidelines demonstrates the potential to reduce mortality, implementing these guidelines sustainably proves challenging in health systems with overtaxed staff and reduced resources.^10,11^ The effective delivery of paediatric emergency care involves a multifaceted interplay encompassing community perceptions, organizational dynamics, management approaches, and the awareness and adherence of individual health workers to best practices. Vital components for improving healthcare quality include establishing credible governance strategies, local support systems, and robust health professional training programs.^12^

Nevertheless, we propose that improved availability of updated, evidence-based guidelines would facilitate sustained implementation. Additionally, utilization of educational techniques, like simulation, would improve health workers’ knowledge and comfort with practice guidelines, thus improving their management of acutely ill children.^13^ Thus, easily available standard protocols that are optimized for use in low- and middle-income countries are needed. Guidelines should be routinely reviewed and updated by their publishing institution to ensure the inclusion of latest available evidence. These core guidelines can then be adapted based on local and facility level needs, allowing for continued dissemination of the most up to date management strategies based on individual department needs.

In conclusion, the significance of updated, evidence-based guidelines cannot be overstated in bolstering paediatric emergency care in low-resource settings. We acknowledge that successful implementation requires a multifaceted approach, addressing resource limitations, navigating complex healthcare landscapes, and fostering adaptive strategies tailored to local contexts. Ensuring updated guidelines is thus not a standalone solution but represents a critical tool in the much-needed arsenal aimed at enhancing the management of acutely ill children in low-income settings. The existing ETAT+ guidelines offer a promising foundation for further protocol development.
